# Chitosan Functionalized with Carboxyl Groups as a Recyclable Biomaterial for the Adsorption of Cu (II) and Zn (II) Ions in Aqueous Media

**DOI:** 10.3390/ijms23042396

**Published:** 2022-02-21

**Authors:** Asmaa M. Abu El-Soad, Giuseppe Lazzara, Mahmoud O. Abd El-Magied, Giuseppe Cavallaro, Jamelah S. Al-Otaibi, M. I. Sayyed, Elena G. Kovaleva

**Affiliations:** 1 Department of Technology of Organic Synthesis, Institute of Chemical Technology, Ural Federal University, Mira St. 19, 620002 Yekaterinburg, Russia; e.g.kovaleva@urfu.ru; 2 Nuclear Materials Authority, El Maadi, Cairo 11381, Egypt; mahmoud_nma@yahoo.com; 3 Dipartimento di Fisica e Chimica, Università degli Studi di Palermo, Viale delle Scienze, Parco d’Orleans II, Ed. 17, 90128 Palermo, Italy; giuseppe.cavallaro@unipa.it; 4 Department of Chemistry, College of Science, Princess Nourah bint Abdulrahman University, Riyadh 11671, Saudi Arabia; jsalotabi@pnu.edu.sa; 5 Department of Physics, Faculty of Science, Isra University, Amman 11622, Jordan; dr.mabualssayed@gmail.com; 6 Department of Nuclear Medicine Research, Institute for Research and Medical Consultations (IRMC), Imam Abdulrahman bin Faisal University (IAU), Dammam 31441, Saudi Arabia

**Keywords:** biomaterials, adsorption, thermodynamic studies

## Abstract

The modification of chitosan represents a challenging task in obtaining biopolymeric materials with enhanced removal capacity for heavy metals. In the present work, the adsorption characteristics of chitosan modified with carboxyl groups (CTS-CAA) towards copper (II) and zinc (II) ions have been tested. The efficacy of the synthesis of CTS-CAA has been evaluated by studying various properties of the modified chitosan. Specifically, the functionalized chitosan has been characterized by using several techniques, including thermal analyses (differential scanning calorimetry and thermogravimetry), spectroscopies (FT-IR, XRD), elemental analysis, and scanning electron microscopy. The kinetics and the adsorption isotherms of CTS-CAA towards both Cu (II) and Zn (II) have been determined in the aqueous solvent under variable pH. The obtained results have been analyzed by using different adsorption models. In addition, the experiments have been conducted at variable temperatures to explore the thermodynamics of the adsorption process. The regeneration of CTS-CAA has been investigated by studying the desorption process using different eluents. This paper reports an efficient protocol to synthesize chitosan-based material perspective as regenerative adsorbents for heavy metals.

## 1. Introduction

Water pollution is frequently due to the presence of heavy metals [1], which include mercury, lead, cadmium, nickel, arsenic, copper, and zinc. Industrial effluents can cause the pollution of water resources since they contain large amounts of heavy metals. Despite the importance of copper for industrial and agricultural processes, it is highly toxic for drinking water. The excessive ingestion of copper causes substantial problems, such as high blood pressure, damage of the kidney and liver, respiratory diseases and convulsions [2,3]. Similarly, zinc is important for regulating many biochemical processes and physiological functions of living tissue, but its excess amount can cause many health problems like stomach sickness, skin irritations, cramps, and anemia [4]. In the last few decades, scientists have given great attention to the removal of these pollutants from the water due to their harmful effect on human life. Several techniques have been improved to remove those pollutants from aqueous solutions, such as solid–liquid extraction, membrane technologies, electrochemical treatment, ion exchange, chemical precipitation, reverse osmosis, liquid–liquid extraction, and ultrafiltration [5,6]. Solid–liquid extraction is considered a promising procedure for the separation and preconcentration of heavy metals since it overcomes the disadvantages of other techniques and provides many features, such as a high enrichment factor, lower consumption of reagents, easy miscibility of solvents, and sustainability [7]. Furthermore, the adsorbent can be reused due to its regeneration from the system by desorption of the metals from its surface [8]. The sorption of copper and zinc has been studied by utilizing several adsorbents, such as activated carbon, bentonite, garden grass, chabazite (natural zeolite), rice husk ash, biomatrix from rice husk, lignite [9,10,11,12,13,14,15], humic acid, cellulose, modified palygorskite, modified Spirulina platensis, calcined horn core [16,17,18], diatomite, flax shive, waste activated sludge biosolid, and multiwall carbon nanotubes (CNTs) oxidized with nitric acid [19,20,21,22]. Nowadays, many researchers focus on the use of biopolymers for the treatment of drinking water since these materials are biodegradable and can be obtained from renewable sources [23]. Among the biopolymers, chitin and chitosan are very promising for wastewater remediation [24]. Chitosan as a biopolymer can be gained by the alkaline deacetylation of chitin. Wastewater can be purified from the heavy metals by using chitosan due to its chelating properties. Chitosan possesses amino and hydroxyl groups, which can be easily incorporated into the formation of metal–chitosan complexes, enhancing its use in adsorption processes. The ability of chitosan to form complexes with metals depends on several parameters, including deacetylation degree, the length of the polymer chain, and crystallinity [25]. As a result, the heavy metal ions can bind to the chitosan-based matrices as a result of the basic strength of the primary amine groups [26]. As evidenced in recent reviews [27,28,29], chitosan can be used for several biocompatible applications in aqueous, gel, and solid phases due to its properties that are influenced by environmental variables such as pH, ionic strength, and electric field. The combination of chitosan with inorganic nanoparticles was explored for the fabrication of drug delivery systems [30,31,32], cleaning gels [33,34], and functional films [35,36].

This work aims to functionalize chitosan with carboxylate groups by using a novel synthesis protocol, which includes cross-linking by glutaraldehyde and epichlorohydrin, further modification through diethylene triamine, and the addition of monochloroacetic acid. Due to its promising chelation properties, the functionalized chitosan (CTS-CAA) has been tested as an adsorbent towards copper and zinc ions in aqueous solutions under variable conditions in terms of pH and temperature. Furthermore, the reusability of (CTS-CAA) has been evaluated by studying the desorption process of heavy metals using several eluents.

## 2. Experimental Section

### 2.1. Materials

Chitosan was gained from Mahtani Chitosan PVT. Ltd., Gujarat, India, and its degree of deacetylation was 86%, and glutaraldehyde 50% (GL) was provided by Acros Organics, Geel, Belgium. Diethylenetriamine (DET) was supplied by Shanghai Chemical Reagent Co. Ltd., Shanghai, China. Epichlorohydrin (EC) and monochloroacetic acid (CAA) were supplied by Alfa Aesar company, New York, NY, USA. ZnCl_2_.3H_2_O and CuCl_2_.H_2_O were products of Alfa Aesar. All other chemicals were Prelab products. All the solutions utilized in this investigation were designed with demineralized water.

### 2.2. Synthesis of Chitosan Modified by Monochloroacetic Acid (CTS-CAA)

The modification of chitosan was carried out in four consecutive steps as reported below.

1. The cross-linking of chitosan by glutaraldehyde to obtain (CTS-GL). To this purpose, 4 g of chitosan were breaking down into acetic acid solution (200 mL; 5 M). The solution was magnetically stirred until it became homogenous. Then, we added 2 mL of glutaraldehyde solution (50%). The mixture was kept at gentle heat (40 °C) for 2 h. The obtained material was filtered and was washed using ethanol and allowed to dry for using in the second step. The obtained material was reported as (CTS-GL) [37,38].
2. The reaction of (CTS-GL) with epichlorohydrin to yield (CTS-EC). 15 mL of epichlorohydrin were mixed with 200 mL of (acetone/water; volume ratio 1:1). Then, 4 g of (CTS-GL) were added, which was previously mixed in 150 mL of isopropanol for 30 min under stirring conditions. The obtained mixture allowed for refluxing for 24 h at 65 °C. The obtained material was filtered and was washed using ethanol and allowed to dry for using in the third step. This material was reported as (CTS-EC) [39,40].
3. The reaction of (CTS-EC) with diethylene triamine to obtain (CTS-DET). For this purpose, 4 g of (CTS-EC) were mixed in 200 mL of (ethanol/water; volume ratio 1:1) under stirring at 25 °C. Then, 10 mL of diethylene triamine were introduced to this reaction media, and the mixture was allowed to react at 65 °C for 12 h under stirring. The obtained material was filtered and was washed using ethanol and allowed to dry for using in the fourth step. The resulting material was reported as (CTS-DET).
4. The reaction of (CTS-DET) with monochloroacetic acid to synthesize (CTS-CAA). In this step, 4 g of (CTS-DET) were mixed in 55 mL of solvent (44 mL of isopropyl alcohol and 11 mL of H_2_O). Then, 6 g of monochloroacetic acid (previously dissolved in 8 mL of isopropanol) were slowly mixed with the media components, and the reaction was continuously run in a water bath under stirring at 60 °C for 5 h. The reaction was stopped by adding 150 mL of diluted ethanol (60%). The obtained material was filtered and was washed using ethanol and allowed to dry for using as an effective sorbent. The product was reported as (CTS-CAA).

### 2.3. Characterization

The elemental composition of unmodified chitosan and (CTS-CAA) samples was determined by an elemental analyzer (2400 CHNS Organic Elemental Analyzer 100 V) for C, H, and N. To determine the functional groups for the modified chitosan sample (CTS-CAA), FTIR was carried out by using the Compact FT-IR Spectrometer ALPHA II in the wavenumber range of (4500–600) cm^−1^.

The pH_PZC_ was estimated via the pH-drift method: 30 mg of (CTS-CAA) was dropped into 15 mL of a 0.1 M NaCl at different initial pH. After 24 h of contact, the equilibrium pH (pH_eq_) was measured by using the Mettler Toledo pH meter. The difference between the initial and final pH values was plotted against the pH_i_. The point at which (pH_f_–pH_i_) = 0 gives the value of the pH_PZC_ [41]. The morphology of the samples was investigated by scanning electron microscope using a Carl Zeiss EVO LS 10 Device. Thermogravimetry-differential scanning calorimetry (TG-DSC) was conducted in the air using a NETZSCH STA449F3 thermal analyzer and the heating rate was fixed at 20/10 K/min. XRD patterns were obtained by using X-ray diffractometer Panalytical X ‘PERT PRO MRD, equipped with an anticathode of Cu Kα. The diffractograms were recorded at 2Ɵ between 10 to 70° using a scanning rate of 0.09°/s and a step size of 0.05°.

### 2.4. Sorption and Desorption Experiments

The adsorption of both copper and zinc ions on the functionalized chitosan was performed by mixing 25 mg of the (CTS-CAA) sample with 50 mL of an aqueous solution containing Cu (II) or Zn (II) with an initial concentration (C_0_) of 1.573 and 1.529 mmol/L for copper and zinc, respectively. The pH of the aqueous medium was systematically varied in the range between 2 and 6.2. The suspensions were kept under stirring for 120 min. Finally, the residual metal ion concentration (C_eq_) was determined by using cuprizone and pyridylazo naphthol (PAN) spectrophotometric methods for copper and zinc ions, respectively [42]. The adsorption capacity (Q_eq_) expressed as mmol of metal per g of (CTS-CAA) was evaluated based on this Equation: Q_eq_ = (C_0_ − C_eq_) × V/M where V is the volume of the metal ion aqueous solution, and M is the mass of (CTS-CAA).

To examine the kinetics of the adsorption processes, 25 mg of (CTS-CAA) sample were kept within 50 mL of an aqueous solution containing the ions (C_0_ = 1.573 and 1.529 mmol/L for copper and zinc, respectively) at variable times (in the range of 10–240 min) under the pH of 3.5. The initial concentration effect of the ions in the adsorption mechanism was tested by systematically changing C_0_ within this range (0.393–5.507 mmol/L for copper) and (0.382–5.353 mmol/L for zinc), and (120 min) was the contact time between the adsorbent and the solution containing the heavy metals. These results allowed us to determine the adsorption isotherms at variable temperatures (298, 304, 309, and 315 K). The desorption experiments were done by mixing a suitable quantity of (CTS-CAA) with a sorbent dosage equal to 0.5 gL^−1^ at the equilibrium conditions, then the metals were regenerated from the sorbent using an appropriate eluent. The eluent and the (CTS-CAA) sample loaded with the metal ions were kept in contact under stirring for 2 h at room temperature. Different eluents like HCl, H_2_SO_4_, HNO_3_, urea, EDTA, and NH_4_Cl were quizzed for the desorption of both copper and zinc metal ions from the (CTS-CAA) surface. It was found that using 0.5 M of HNO_3_ and 0.2 M of urea for copper and zinc, respectively, can greatly regenerate the metals from the sorbent surface.

## 3. Results and Discussion

### 3.1. Characterization of Chitosan Modified by Monochloroacetic Acid

Table 1 reports the elemental analysis for chitosan, chitosan after its cross-linking by glutaraldehyde (CTS-GL), (CTS-GL) after its reaction with epichlorohydrin (CTS-EC), (CTS-EC) after its reaction with diethylenetriamine (CTS-DET), and (CTS-DET) after its reaction with monochloroacetic acid (CTS-CAA).

Concerning (CTS-EC), the chlorination of chitosan by epichlorohydrin was confirmed by the decrease of carbon, hydrogen, and nitrogen contents. The successful grafting of diethylenetriamine on the (CTS-EC) sample was evidenced by the significant enhancements of carbon, hydrogen, and nitrogen contents detected in the (CTS-DET) sample. The successful grafting of monochloroacetic acid on the (CTS-DET) sample was highlighted by the significant decrease of carbon, hydrogen, and nitrogen amounts estimated in the (CTS-CAA) sample.

**Figure 1:**
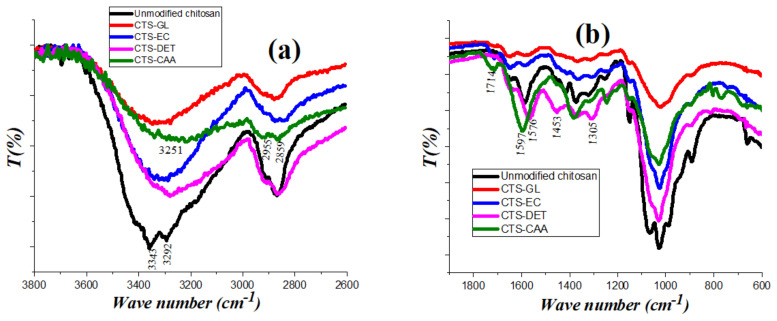
FT-IR spectra of (unmodified chitosan), (CTS-GL), (CTS-EC), (CTS-DET), and (CTS-CAA) [wavenumber range limited to 3800–2600 cm^−1^] (**a**), [wavenumber range limited to 1900–600 cm^−1^] (**b**).

Figure 1a,b displays FT-IR spectra of (unmodified chitosan), (CTS-GL), (CTS-EC), (CTS-DET), and (CTS-CAA) at a limited wavenumber range, while the FT-IR spectra of these samples within the full wavenumber range is provided in the supplementary file (Figure S1). The analysis of the FT-IR spectra provided reliable information on the efficacy of the synthesis procedure used for the chitosan modification.

The FT-IR spectrum of unmodified chitosan showed two sharp bands centered at 3343 and 3292 cm^−1^ due to the asymmetric and the symmetric N-H stretching, respectively. These broader signals are characteristic for primary amines in the region 3400–3250 cm^−1^, hiding the broadband of O-H groups. A band centered at 1564 cm^−1^ was observed, which is related to the N-H bending occurring in the region 1650–1580 cm^−1^ for primary amines. In the spectrum of CTS-GLA, broadband at 3378 cm^−1^ was detected due to the alcohol O-H stretching in the range between 3200–3400 cm^−1^. Accordingly, it can be stated that the two sharp bands of the N-H stretching have disappeared, highlighting the actual cross-linking of NH_2_ groups by glutaraldehyde molecules. The additional bands were noticed within the area of 2951–2830 cm^−1^ due to the stretching vibration of the CH_2_ groups, which are present in glutaraldehyde molecules. The spectrum of CTS-DET showed the presence of sharp bands centered at 3274, 1576, and 1453 cm^−1^ (due to the N-H stretching of secondary amines and the N-H bending of primary amines, respectively), confirming the reaction between CTS-EC and diethylenetriamine. In the spectrum of CTS-CAA, an additional sharp peak was detected at 1714 cm^−1^, which is related to the stretching of C=O of carboxylic acids, evidencing the actual reaction between CTS-DET and monochloroacetic acid. Moreover, CTS-CAA exhibited two weak sharp bands at 2859 and 2955 cm^−1^ (caused by the CH_2_ stretching vibration of -CH_2_COOH) and the broadband in the region between 3358 and 3171 cm^−1^, which are generated by the stretching of N-H and O-H groups. The shift of the N-H band from 1576 cm^−1^ to 1597 cm^−1^ is due to conjugation.

**Figure 2:**
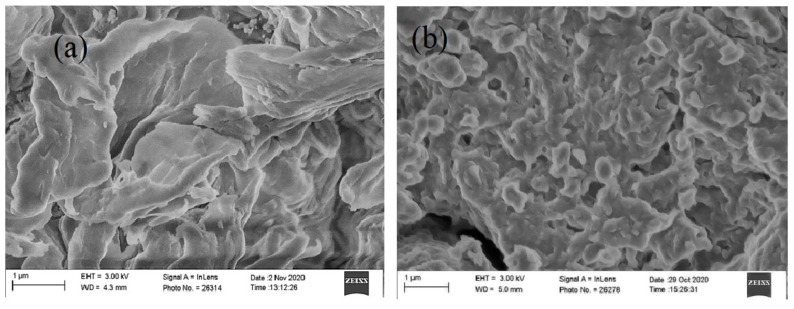
Scanning electron microscopy images for unmodified chitosan (**a**) and CTS-CAA (**b**).

As displayed in SEM images (Figure 2), the chitosan modification with carboxyl groups (CTS-CAA) affected the surface morphology of the biopolymer.

**Figure 3:**
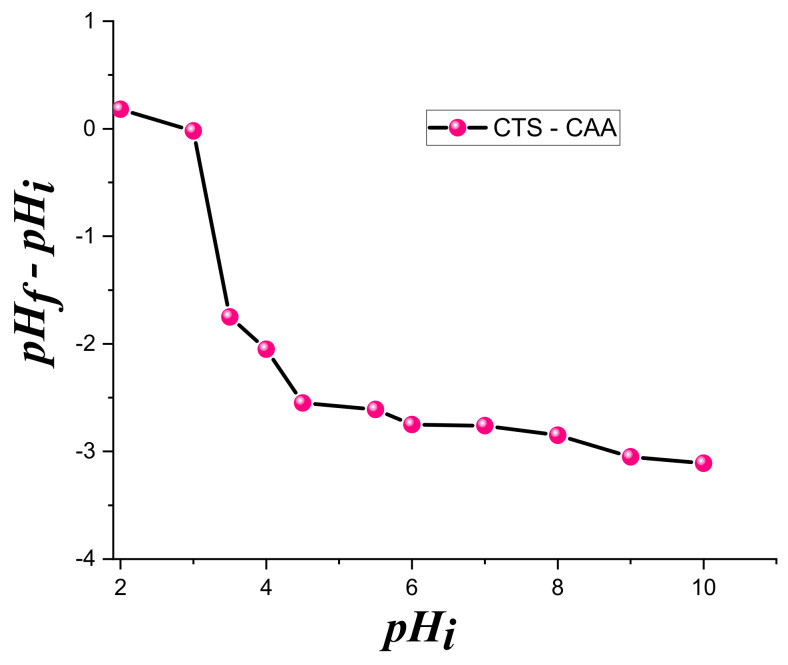
The plot of estimating the pH_pzc_ for CTS-CAA in a 0.1 M NaCl solution based on titration method.

The titration curve of pH_PZC_ estimation for (CTS-CAA) sample is presented in Figure 3. The obtained results can be useful to investigate the effect of the modification on the surface charge of chitosan.

According to Figure 3, we estimated that the isoelectric point of (CTS-CAA) occurred at pH equal to ca. 3. The surface of (CTS-CAA) was negatively charged at a pH larger than 3 because of the deprotonation of carboxyl groups. Therefore, chelation processes for the heavy metal adsorption can be expected within this investigated pH range. Namely, copper and zinc cations can be adsorbed at pH values greater than pH_pzc_.

**Figure 4:**
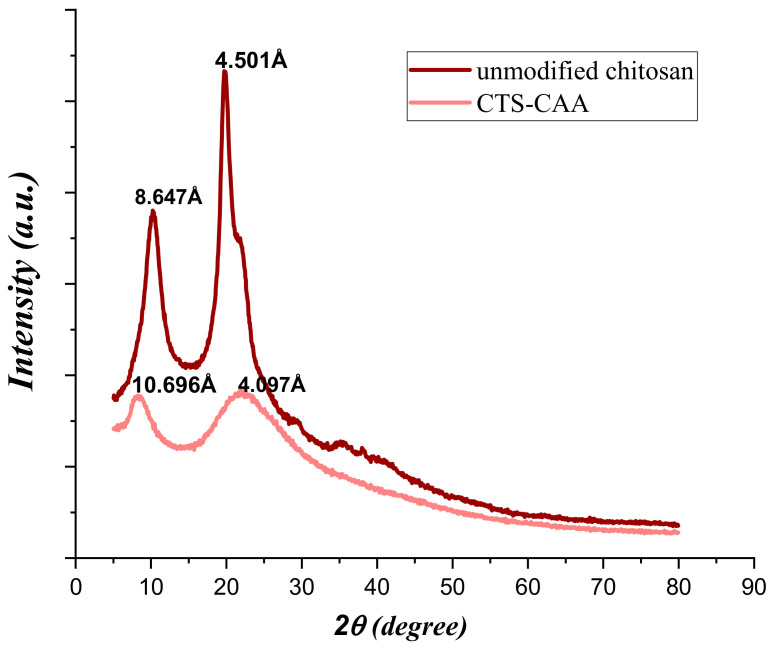
XRD diffractograms of unmodified chitosan and CTS-CAA.

Figure 4 compares the XRD diffractograms of chitosan before and after the functionalization with carboxyl groups.

Unmodified chitosan evidenced significant peaks at 2θ equals to 10.22 and 19.707°, while the main XRD peaks for the CTS-CAA sample were observed at 2θ equals to 8.259 and 22.145°. On this basis, it can be stated that the functionalization of chitosan caused a shift of the first peak to a lower 2θ value, while the opposite effect was detected for the second peak. These observations highlight that the interplanar spacing in the chitosan polymer was strongly changed after the modification procedure. The broadening of the reflection peaks in the CTS-CAA sample may be due to the high degree of chitosan functionalization.

**Figure 5:**
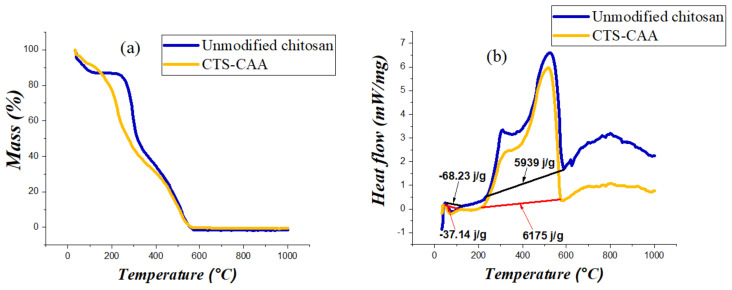
TGA (**a**) and DSC (**b**) curves for unmodified chitosan and CTS-CAA.

The impact of modifying the surface on the thermal properties of chitosan was investigated by TGA and DSC methods (Figure 5).

Figure 5a displays the TGA curves of both unmodified chitosan and CTS-CAA. The decomposition of both materials is complete at ca. 600 °C. Unmodified chitosan exhibited two well-defined mass losses in the ranges 25–120 °C and 240–580 °C. The first mass loss (12.4%) is related to the moisture content of the polymer, while the second mass change (87.6%) could be assigned to the thermal degradation of the organic moieties. On the other hand, the estimated moisture loss of CTS-CAA equals 9.53%. Accordingly, it can be stated that the chitosan modification generated a reduction in the hydrophilicity of the biopolymer. Compared to unmodified chitosan, the degradation of the organic moieties occurred over a larger interval (ca. 180–580 °C) due to the addition of the carboxyl moieties.

As displayed in Figure 5b, the DSC curves of both unmodified chitosan and CTS-CAA present an exothermic peak centered at ca 520 °C that can be attributed to the degradation of the polymeric chains. There has been a noticeable enlargement in the endothermic peak area of (CTS-CAA) in comparison with that obtained for unmodified chitosan. This could be assigned to the fact that the hydrogen bonds between the amino groups and water molecules in the case of unmodified chitosan are weaker than those formed with the terminal carboxyl groups in the case of (CTS-CAA), hence the temperature required to remove those water molecules will be greater in the case of (CTS-CAA) than in the case of unmodified chitosan.

### 3.2. Mechanism of Adsorption of Copper and Zinc Ions on CTS-CAA

**Figure 6:**
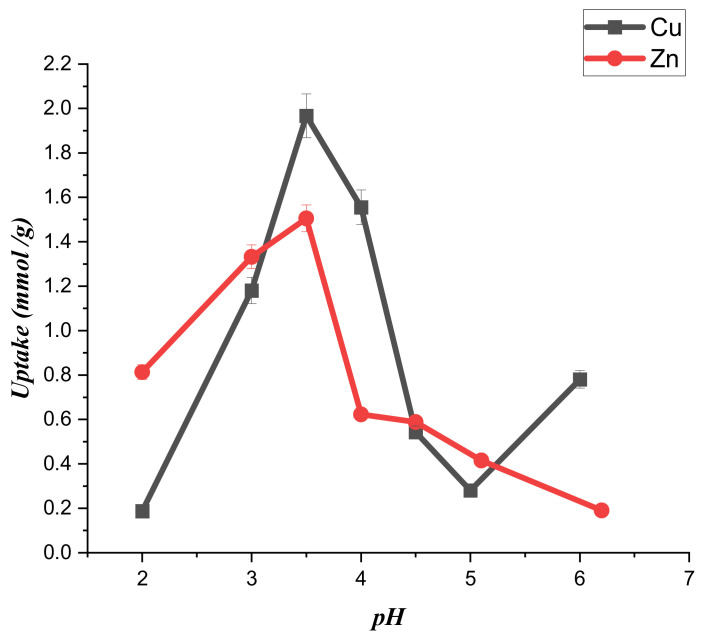
pH effect on the sorption capacity of (CTS-CAA) sorbent towards both copper and zinc ions.

The pH conditions affect the speciation of the metal and control the adsorbent’s surface charge. As a result, the pH may control the entire adsorption system. According to the data in Figure 3, (CTS-CAA) possesses a negative surface charge for pH > 3. Therefore, the modified chitosan is negatively charged for the pH conditions (ranging between 3.5 and 6) used in the adsorption experiments. Namely, the carboxylate groups on the surface of (CTS-CAA) are deprotonated within the studied pH range, while the metal ions are positively charged. On this basis, we could predict that electrostatic attractions between the metal ions and the negatively charged surface of the modified chitosan at a pH range between 3.5 and 6. The adsorption capacity could be imputed to the chelation mechanism between metal (Cu, Zn) ions and the –(COO)^−^ of (CTS-CAA). In contrast, the protonation of the (CTS-CAA) amino groups is expected at a pH < 2.5, reducing the adsorption capacity of the modified chitosan towards the heavy metals. These considerations were confirmed by the experimental results on the uptake capacity of the (CTS-CAA) sample towards both copper and zinc ions (Figure 6). It can be estimated that the greatest uptake for both metal ions occurred at pH = 3.5. In particular, the uptake values are 1.96 ± 0.05 mmol/g and 1.505 ± 0.04 mmol/g for copper and zinc metal ions, respectively. 

### 3.3. Kinetics of the Metal Ions Adsorption on Modified Chitosan

**Figure 7:**
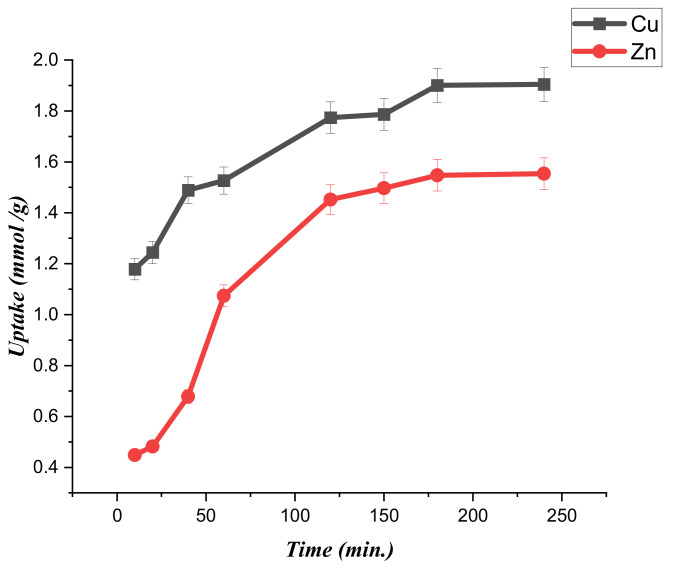
Time effect on the uptake capacity of (CTS-CAA) sorbent towards both copper and zinc ions.

To esteem the kinetic aspects of the adsorption processes, the uptake capacity of the (CTS-CAA) sample towards the heavy metal ions was determined at a variable time within the range (10–240) of minutes. The adsorption experiments were performed under isothermal conditions (temperature was set at 25 °C) under pH = 3.5. Figure 7 displays the dependences of the metal ions uptake on time.

It was observed that the modified chitosan presents an efficient adsorption capacity (larger than 60% of the uptake at saturation) after 40 and 60 min for copper and zinc ions, respectively. The saturation of the adsorption sites of the (CTS-CAA) sample generated uptake values of ca. 1.9 ± 0.035 and 1.5 ± 0.04 mmol/g for Cu (II) and Zn (II) ions, respectively.

The profiles presented in Figure 7 were analyzed using numerous kinetic models to estimate the rate-limiting step for the adsorption processes. Specifically, we employed the pseudo.first.order (Lagergren paradigm, Equation (1), pseudo.second.order (Ho and Mckay paradigm, Equation (2) [43], Intra-particle diffusion (Equation 3), Dumwald-Wagner paradigm (Equation (4)), Elovich’s paradigm (Equation (5)), and Bangham paradigm (Equation (6)) were employed [44].

(1) $$\log\left(q_{e}-q_{t}\right)=\log q_{1}-(\frac{K_{1}}{2.303})t\;$$

(2) $$\frac{t}{q_{t}}=\frac{1}{K_{2}q_{2}^{2}}+\frac{1}{q_{2}}t\;$$

(3) $$q_{t}=Z+K_{i}t^{0.5}\;$$

(4) $$\;\log\left(1-\left(q_{t}/q_{e}\right)\right)=\left(-\frac{K_{\mathrm{fd}}}{2.303}\right)t\;$$

(5) $$q_{t}=\left(\frac{1}{B}\right)\ln\left(\mathrm{aB}\right)+\left(\frac{1}{B}\right)\ln\left(t\right)$$

(6) $$\;\log\;\left(\frac{C_{0}}{C_{0}-{\mathrm{mq}}_{t}}\right)=\log\left(\frac{{\mathrm{mK}}_{\gamma }}{2.303\;V}\right)+\gamma \log t$$

In the Equations (1)–(6), q_e_ and q_t_ represent the metal ions quantities adsorbed on (CTS-CAA) (mmol_(Cu, Zn)_.g^−1^ _CTS-CAA_) at the equilibrium and variable time t (min), respectively. The parameters q_1_ and q_2_ are the calculated metal uptakes according to the pseudo.first.order (Equation (1)) and the pseudo.second.order models (Equation (2)), respectively, while K_1_ is the pseudo.first.order rate constant (min^−1^) and K_2_ is the pseudo.second.order rate constant (g.mmol^−1^.min^−1^). Equation (3) presents K_i_ [intra-particle diffusion rate constant expressed as mmol.g^−1^.min^−0.5^] and Z (constant expressed as mmol g^−1^) for the intra-particle diffusion model. In Equation (4), K_fd_ is the Dumwald-Wagner rate constant (min^−1^) and the (q_t_/q_e_) ratio is generally reported as F, while Equation (5) contains a and B as Elovich’s equation constants. In Equation (6), K_γ_ and γ are the Bangham constants, C_0_ (mmol L^−1^) is the initial metal ion concentration, m is the weight of (CTS-CAA) per liter of solution (g.L^−1^), V is the volume of metal ion solution (ml). The fitting plots related to the described kinetic models are presented in Figure S2 [supporting information section]. Considering those plots, the adsorption parameters were determined, which are reported in Table S1 (supporting information section).

Considering the R^2^ values (Table S1), we could state that the pseudo.second.order and Elovich’s equation are the more accurate fitting procedures in the analysis of the adsorption results for both copper and zinc. On the other hand, the Dumwald-Wagner model did not provide reliable fitting parameters.

### 3.4. Adsorption Isotherms of the Metal Ions Adsorption on Modified Chitosan

**Figure 8:**
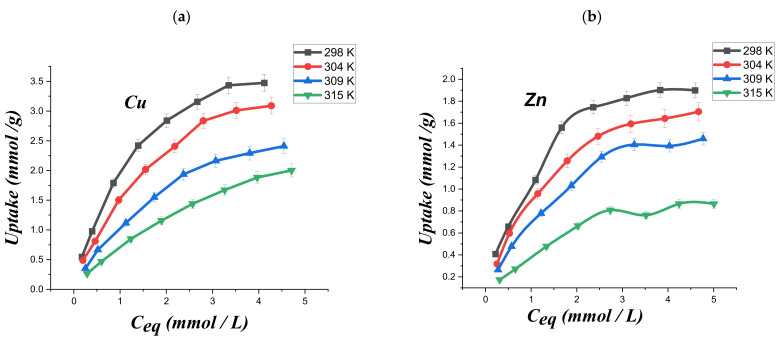
Adsorption isotherms for copper (**a**) and zinc (**b**) ions.

Figure 8 displays the adsorption isotherms of both metal ions on modified chitosan at variable temperatures.

As a general result, the increase in the temperature reduced the metal ions uptake on the modified chitosan (Figure 8). The adsorption isotherms at different temperatures were analyzed using several fitting models, including Langmuir, Freundlich, Dubinin–Radushkevich, and Temkin.

Based on the Langmuir model, the equilibrium adsorption amount of ions (q_e_) can be expressed by Equation (7) [45]:

(7) $${\;q}_{e}=\frac{q_{x.{\;K}_{l\;}.C_{\mathrm{eq}}}}{\left(1+K_{l}{\;C}_{\mathrm{eq}}\right)}$$

Being that q_x_ is the greatest sorption capacity of (Cu, Zn) on (CTS-CAA) sorbent, and C_eq_ is the metal ion concentration in solution at equilibrium.

Equation (8) reports the Langmuir model in the linear form

(8) $$\frac{C_{\mathrm{eq}}}{q_{e}}=\frac{C_{\mathrm{eq}}}{q_{x}}+\frac{1}{K_{l}q_{x}}$$

**Figure 9:**
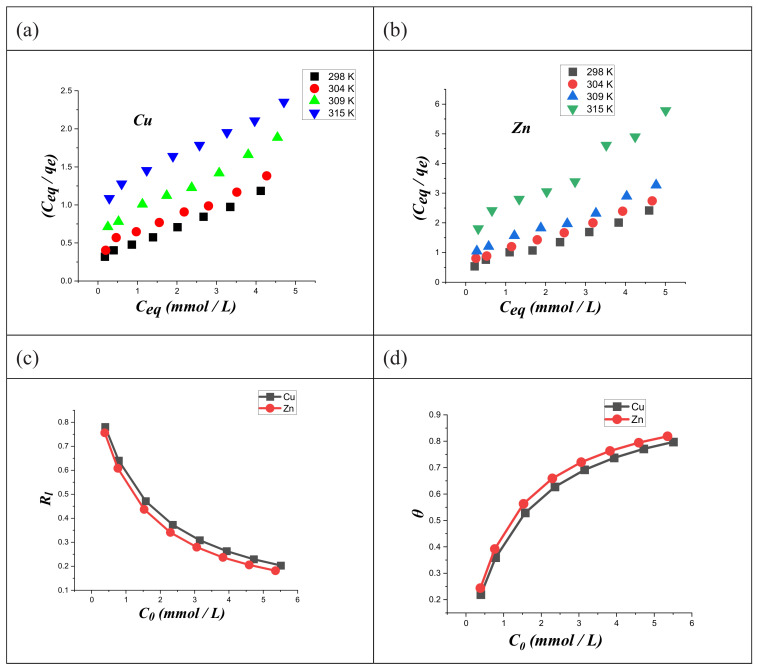
Langmuir model for copper (**a**) and zinc (**b**) ions. Dimensionless separation factor (**c**) and surface coverage (**d**) for the adsorption of both metal ions on modified chitosan.

The linearity predicted by Equation (8) was observed in the (C_eq_/q_e_) vs. C_eq_ plots (Figure 9a,b) for both copper and zinc ions at the different investigated temperatures. On this basis, we could assert that the Langmuir model is appropriate for the description of the adsorption process. Accordingly, we can conclude that (CTS-CAA) possesses a homogeneous surface with energetically equal adsorption sites.

The estimated K_l_ values were included in Equation (9) to determine the dimensionless separation factor (R_l_), which provides information on the affinity between the metal (Cu, Zn) ions and the (CTS-CAA) sorbent surface.

(9) $$R_{l}=\frac{1}{1+K_{l}C_{0}}\;$$

Figure 9c displays the R_l_ vs. C_0_ plots of both copper and zinc. We observed exponential decreasing trends with R_l_ ranging between 0.8 and 0.1. In particular, the R_l_ intervals are 0.78–0.20 and 0.75–0.18 for copper and zinc, respectively. These results highlight the suitability of (CTS-CAA) as an adsorbent material for both metals. The K_l_ values were used to calculate the surface coverage percentage (θ) of the (CTS-CAA) sorbent by using Equation (10):

(10) $$\theta =\frac{K_{l\;}{\;c}_{0}}{1+K_{l\;}{\;c}_{0}}\;$$

As shown in Figure 9d, an increasing θ vs. C_0_ relation for both copper and zinc was detected. The estimated θ value equals ca. 0.8 at the highest C_0_ for both metals.

The Langmuir fitting parameters obtained for the adsorption experiments at variable temperatures are collected in Table 2 and Table 3.

Based on the Van’t Hoff approach [46], we calculated the thermodynamic parameters for the adsorption process of the metal ions on (CTS-CAA) material. In detail, the K_l_ values at variable temperatures are related to the enthalpy (∆H°) and entropy (∆S°) variations according to Equation (11) [47]:

(11) $${\;\mathrm{lnK}}_{l}=\frac{-∆H^{{}^{\circ}}}{\mathrm{RT}}+\frac{∆S^{{}^{\circ}}}{R}$$

From Figure S3 [supporting information section], the experimental ln K_l_ vs. (1/T) plots, shows linear increasing trends, which can be analyzed by Equation (11) to determine ∆H° and ∆S° of the adsorption processes.

The calculated ∆H° values are −48.28 ± 0.2 and −25.83 ± 0.15 (kJ/mol) for Cu (II) and Zn (II), respectively. On the other hand, we determined ∆S° values equal to −164.44 ± 0.2 and −88.33 ± 0.15 J/(K.mol) for copper and zinc, respectively. The corresponding Gibbs free energies (∆G°) at different temperatures can be determined as:

(12) $$∆G^{{}^{\circ}}=∆H^{{}^{\circ}}-T∆S^{{}^{\circ}}\;$$

The obtained ∆G° values are presented in Table 2 and Table 3.

The values of ∆G° (KJ/mol) lie in the range of (0.719–3.515) ± 0.2 and (0.493–1.995) ± 0.15 for copper and zinc, respectively, at a temperature range (298–315) ± 1 K. At the elaborated temperatures, it could be understood that the value of │∆H°│ < │T∆S°│. Based on this, it can be signalized that the adsorption process is commanded by entropic rather than enthalpic changes. The negative values of ∆S ° at different temperatures indicate less degree of randomness for the sorption of (Cu, Zn) on (CTS-CAA), while the negative values of ∆H° at variety of temperature change signalize the exothermic feature of the sorption of (Cu, Zn) on (CTS-CAA). The values of ∆S°, (∆G°), and ∆H° for both copper and zinc at different temperatures were displayed in Table 2 and Table 3.

Furthermore, the adsorption isotherms were analyzed through the Freundlich model [48] using the following expression:

(13) $$\log q_{e}=\log K_{f}+\frac{1}{n}{\;\log\;C}_{\mathrm{eq}}$$

The Freundlich plots are presented in Figure S4a,b [supporting information section], while the obtained fitting parameters are reported in Tables S2 and S3 (supporting information section). It should be noted that the Freundlich approach is valid for n values between 0 and 1. According to the data in Tables S2 and S3 (supporting information section), the calculated n values were larger than 1 for both metals. Therefore, it can be concluded that the Freundlich model is not convenient to describe the adsorption process of Cu (II) and Zn (II) on (CTS-CAA).

The adsorption isotherms were fitted using other approaches, including Dubinin–Radushkevich (D–R) and Temkin models.

The Dubinin–Radushkevich (D–R) model could be stipulated by using Equation (14) [49]:

(14) $$\ln q_{e}=\ln Q_{S}-K_{\mathrm{DR}\;}\varepsilon^{2}$$

where Q_s_ is related to the theoretical saturation capacity (mmol/g), K_DR_ (mol^2^ K J^−2^) refers to the free energy change of sorption per mole of adsorbate, and Ɛ is the Polanyi potential, which is related to the temperature by Equation (15):

(15) $$Ɛ=\;\mathrm{RT}\ln\;\left[1+\left(\frac{1}{\mathrm{Ceq}}\right)\right]$$

The mean free energy needed to transfer one mole of ions from the solution to the solid surface of (CTS-CAA) could be enumerated by K_DR_ using Equation (16):

(16) $$E=\frac{1}{{\left(2K_{\mathrm{DR}}\right)}^{0.5}}\;\;$$

Figure S4c,d [supporting information section] shows the ln q_e_ vs. Ɛ^2^ plots as a representation of the Dubinin–Radushkevich model for the adsorption of both copper and zinc on (CTS-CAA), while the fitting parameters are presented in Tables S2 and S3 (supporting information section). As a general result, the obtained E values decrease with the temperature. The interval for the E values is (1.58–1.11) and (1.58–1.29) kJ/mol for copper and zinc, respectively. Being that E values are lower than 8 kJ/mol, we can state that the adsorption of both metals on (CTS-CAA) can be attributed only to the physical sorption mechanism.

As concerns the Temkin isotherm model, q_e_ and C_eq_ are related by the Equation (17) [50]:

(17) $$q_{e}=B\;{\ln\;C}_{\mathrm{eq}}+{B\;\ln\;A}_{T}\;$$

where B is a constant describing the heterogeneity of the (CTS-CAA) surface. The linear plots predicted by the Temkin model are displayed in Figure S4e,f [supporting information section], while the fitting parameters are listed in Tables S2 and S3 (supporting information section). Table 4 displays the adsorption capacities using variety of adsorbents for the separation of Zn (II) and Cu (II) from their aqueous solutions.

### 3.5. Desorption and Regeneration Processes

The regeneration of the (CTS-CAA) sorbent from the samples loaded by the metal ions was studied by performing desorption experiments using different eluents, including 0.5 M HNO_3_, 0.5 M HCl, 0.5 M H_2_SO_4_, 0.2 M EDTA, 0.2 M urea, and 0.2 M NH_4_Cl. The contact time was fixed as 120 min. and the sorbent dosage was fixed at 0.5 g/L for the desorption steps.

The desorption efficiency was evaluated using Equation (18):

(18) $$Desorption\;efficiency=\frac{C\times V}{q_{e}\times m}\times 100\%$$

where *V* is the volume of desorption solution and it is fixed at 50 mL, *q_e_* (mmol/g) is the quantity of Cu^+2^ or Zn^+2^ adsorbed on (CTS-CAA) before applying the desorption processes, and m is the mass of (CTS-CAA) loaded by the metal ions. The successful desorption process was achieved by using 0.5 M of HNO_3_ and 0.2 M of urea for copper and zinc, respectively.

After the first desorption experiments, the (CTS-CAA) material underwent washing with distilled water and was applied for the next cycle of desorption. In total, three successful cycles were performed, and the data obtained for the desorption tests are reported in Table 5. For the first cycle, the sorption efficiency for both copper and zinc was represented as nearly 100% as a reference value, and the sorption capacities for the other cycles were computed relative to the first cycle.

## 4. Conclusions

A novel sorbent derived from chitosan was created and characterized by using elemental analysis, FT-IR, SEM, DSC, TGA, and XRD analysis. The material was functionalized by chloroacetic acid by giving arms of -COOH groups on the surface of the modified sorbent (CTS-CAA). The data reported related to the zeta potential of (CTS-CAA) depicts the negative charge of the carboxyl groups within the pH range of (3.1–10), and thus facilitate the binding between the metal ions and (-COO^−^) on (CTS-CAA) surface. As expected, the pH strongly affects the adsorption capacity of the modulated chitosan. The affinities of Cu (II) and Zn (II) towards the modified chitosan were the largest at pH = 3.5, where strong electrostatic attractions occur. The great affinity of Cu (II) and Zn (II) metal ions towards the modulated chitosan could be imputed to the existence of numerous carboxyl groups onto the chitosan surface after its modification. The sorption capacity was 3.47 mmol Cu g^−1^ and 1.89 mmol Zn g^−1^. Kinetic studies showed fitting of the experimental data with the model provided by Ho and Mckay. On the other hand, the Dumwald–Wagner model did not provide reliable fitting parameters. It could be spotted that the uptake kinetics is rather fast, and more than 60% of the achieved uptake at the saturation is generated after 40 and 60 min for copper and zinc ions, respectively. The adsorption isotherms were fitted using several models. As was detected, the Langmuir isotherm model provided the most reliable results for the description of the adsorption process. Desorption experiments revealed that the modified chitosan can be easily regenerated using proper eluents. Therefore, this synthesized biomaterial might be considered as an efficient and reusable adsorbent for remediation purposes in aqueous media.

## Figures and Tables

**Table 1:** Elemental analysis for chitosan and modified chitosan samples.

Sample	Content (%)		
	C	H	N
Chitosan	40.27	7.91	6.22
(CTS-GL)	43.01	7.23	5.81
(CTS-EC)	37.18	6.17	5.61
(CTS-DET)	43.19	7.34	8.94
(CTS-CAA)	42.54	6.98	7.38

**Table 2:** Langmuir and thermodynamic parameters for copper.

T (K)	Langmuir Parameters			Thermodynamic Parameters		
	q_x_	K_l_	R^2^	∆S° J/K mol	∆H° KJ/mol	∆G° J/mol
298 ± 1	4.77 ± 0.05	0.713 ± 0.01	0.996	−164.44 ± 0.2	−48.28 ± 0.2	719.51 ± 0.2
304 ± 1	4.56 ± 0.05	0.525 ± 0.01	0.987	−164.44 ± 0.2	−48.28 ± 0.2	1706.16 ± 0.2
309 ± 1	3.79 ± 0.05	0.404 ± 0.01	0.992	−164.44 ± 0.2	−48.28 ± 0.2	2528.37 ± 0.2
315 ± 1	3.75 ± 0.05	0.245 ± 0.01	0.991	−164.44 ± 0.2	−48.28 ± 0.2	3515.03 ± 0.2

**Table 3:** Langmuir and thermodynamic parameters for Zinc.

T (K)	Langmuir Parameters			Thermodynamic Parameters		
	q_x_	K_l_	R^2^	∆S° J/K mol	∆H° KJ/mol	∆G° J/mol
298 ± 1	2.47 ± 0.04	0.843 ± 0.015	0.987	−88.327 ± 0.15	−25.82 ± 0.15	493.45 ± 0.15
304 ± 1	2.30 ± 0.04	0.66 ± 0.015	0.996	−88.327 ± 0.15	−25.82 ± 0.15	1023.42 ± 0.15
309 ± 1	2.09 ± 0.04	0.528 ± 0.015	0.986	−88.327 ± 0.15	−25.82 ± 0.15	1465.06 ± 0.15
315 ± 1	1.26 ± 0.04	0.489 ± 0.015	0.972	−88.327 ± 0.15	−25.82 ± 0.15	1995.03 ± 0.15

**Table 4:** Disparity of the adsorption capacities of different adsorbents for the separation of Cu (II) and Zn (II) from their aqueous solutions.

Adsorbent	Adsorption Capacity (mg/g)		
	Cu (II)	Zn (II)	References
Sugar beet pulp	21.1	17.8	[51,52,53]
A biomatrix derived from rice husk	10.8	7.47	[13]
Chitosan–cellulose beads	53.2	-	[54]
Pyromellitic dianhydride modified SCB	77.4	65.0	[55]
Polyaniline graft chitosan	83.30	-	[56]
PEI-RCSA	177.1	110.2	[57]
Waste activated sludge biosolid	-	36.88	[12]
Lewatit SP 112	40.32	64.10	[58]
Lewatit TP 207	68.50	73.00	[59]
CTS-CAA	220.5	124.3	The current study

**Table 5:** Data of three cycles of metal desorption.

Cycle	1		2		3	
Metal	Adsorption (%)	Desorption (%)	Adsorption (%)	Desorption (%)	Adsorption (%)	Desorption (%)
copper	100	80.23	91.56	76.54	88.47	74.36
Zinc	100	84.21	93.42	79.89	89.74	78.89

## Data Availability

All data were included in the manuscript.

## Supplementary Materials

The following supporting information can be downloaded at: https://www.mdpi.com/article/10.3390/ijms23042396/s1.
