# Supplementary material for: Chitosan Functionalized with Carboxyl Groups as a Recyclable Biomaterial for the Adsorption of Cu (II) and Zn (II) Ions in Aqueous Media
Source: Int J Mol Sci. 2022 Feb 21;23(4):2396. doi: 10.3390/ijms23042396 (PMC8875004; doi:10.3390/ijms23042396)
Supplement: Supplementary file 1 [file ijms-23-02396-s001.zip › ijms-1549366-supplementary.pdf]

Electronic supplementary information (ESI)

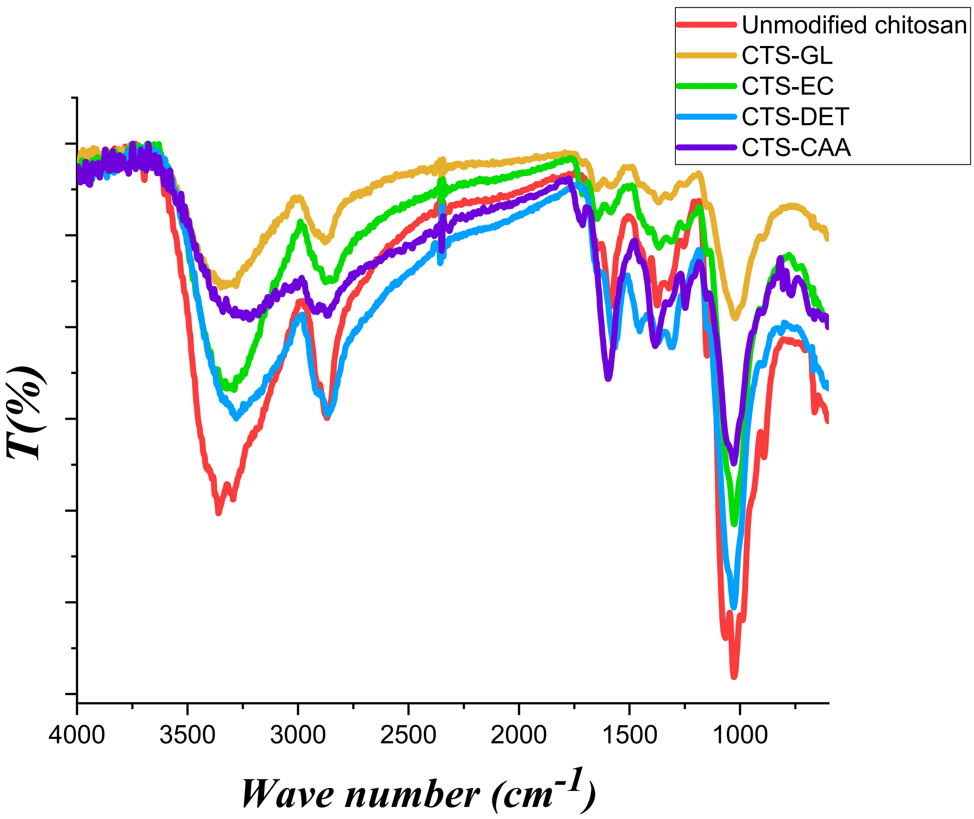

Figure S1. FT-IR spectra of (Unmodified chitosan), (CTS-GL), (CTS-EC), (CTS-DET) and (CTS-CAA) [full wavenumber range].

(a)	(b)
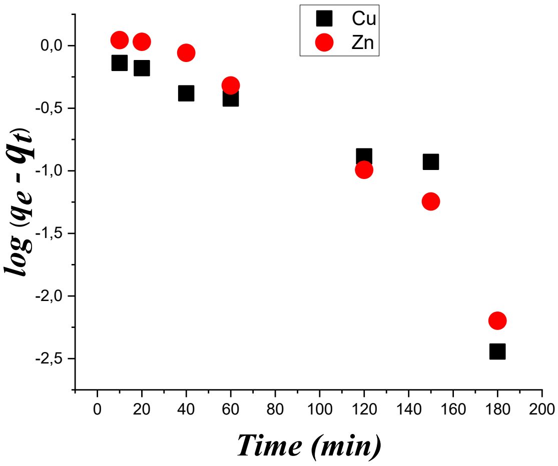	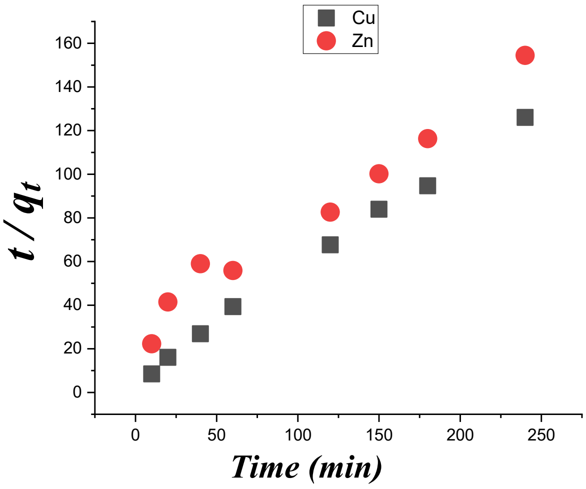
(c)	(d)

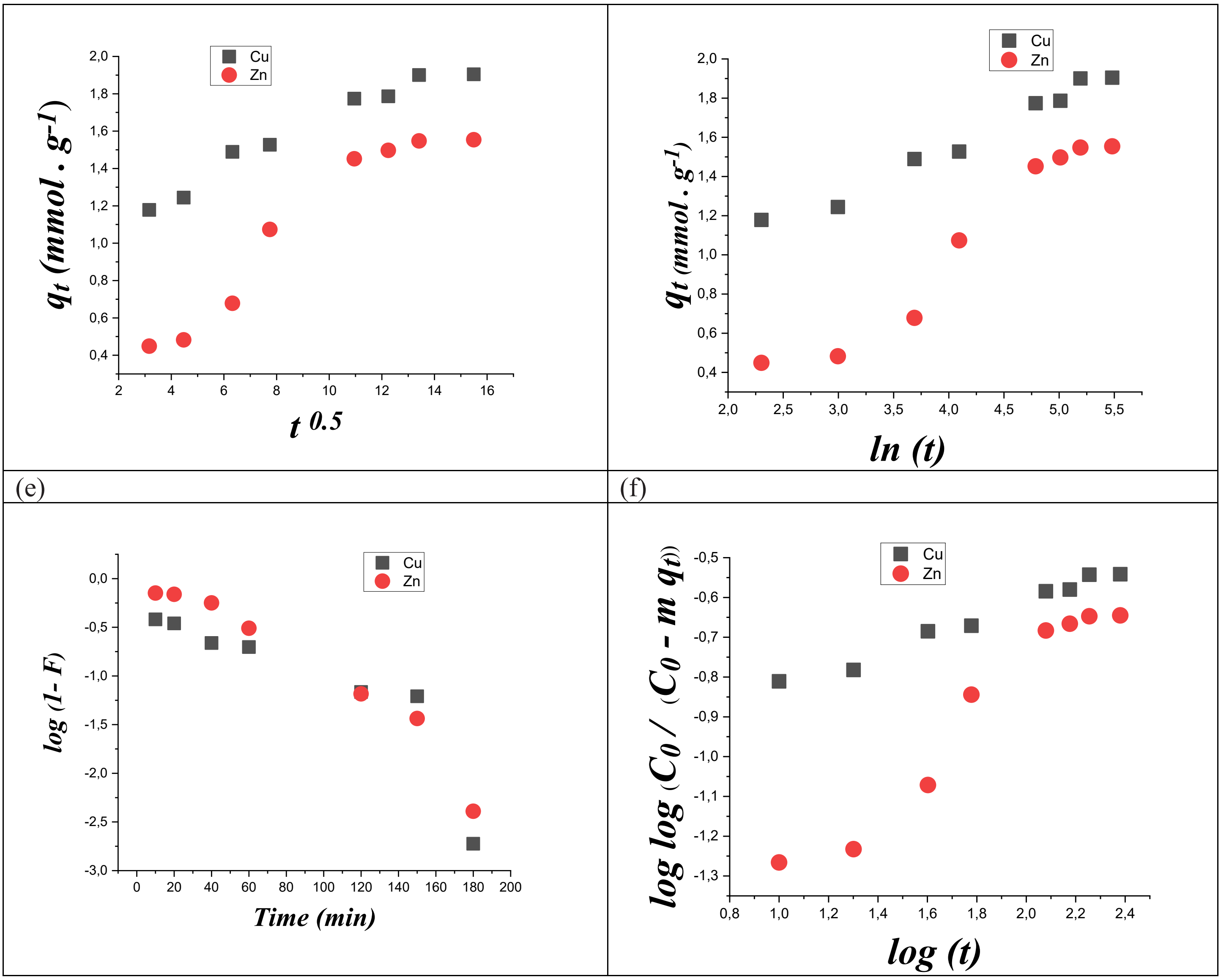

Figure S2. Pseudo-first-order plot with slope equal ($-K_1/2.303$) and the intercept equal log q_1 (a), Pseudo-second-order plot with slope equal ($1/q_2$) and the intercept equal ($1/K_2q_2^2$) (b), Intraparticle diffusion plot with slope equal (K_i) and the intercept equal (Z) (c), Elovich's equation plot with slope equal (1/B) and the intercept equal [(1/B) ln (aB)] (d), Dumwald-Wagner plot with slope equal ($-K_{fd}/2.303$) (e), Bangham plot with slope equal (γ) and intercept equal log (m K_γ /2.303V) (f).

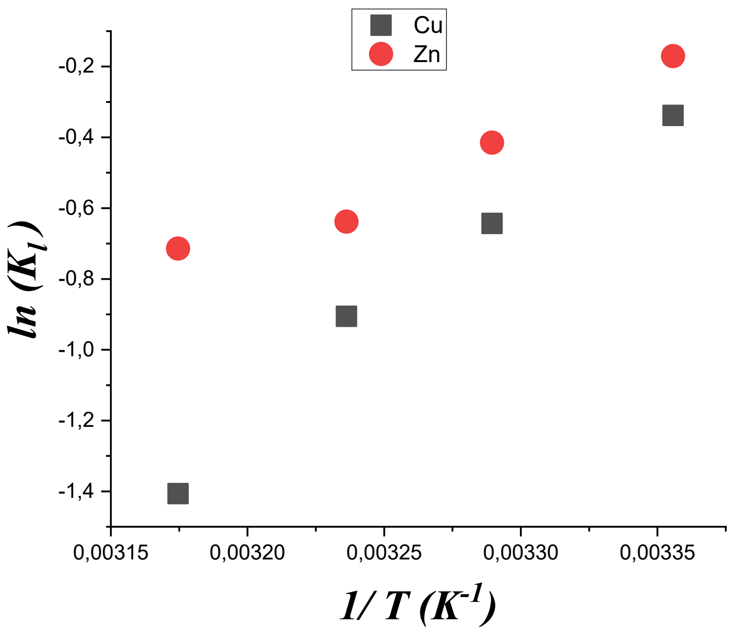

Figure S3. Representation of Van·t Hoff equation for the adsorption of the metal ions on (CTS-CAA).

(a)	(b)
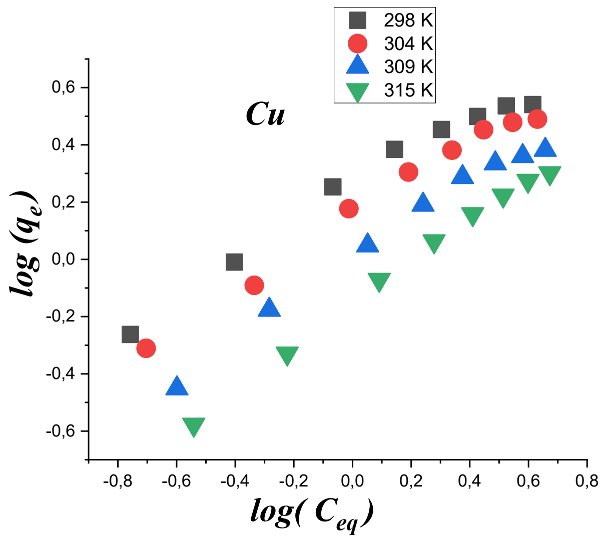	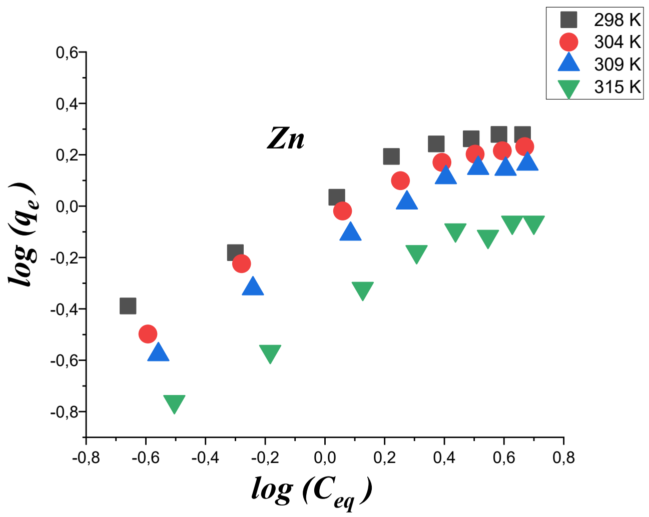
(c)	(d)

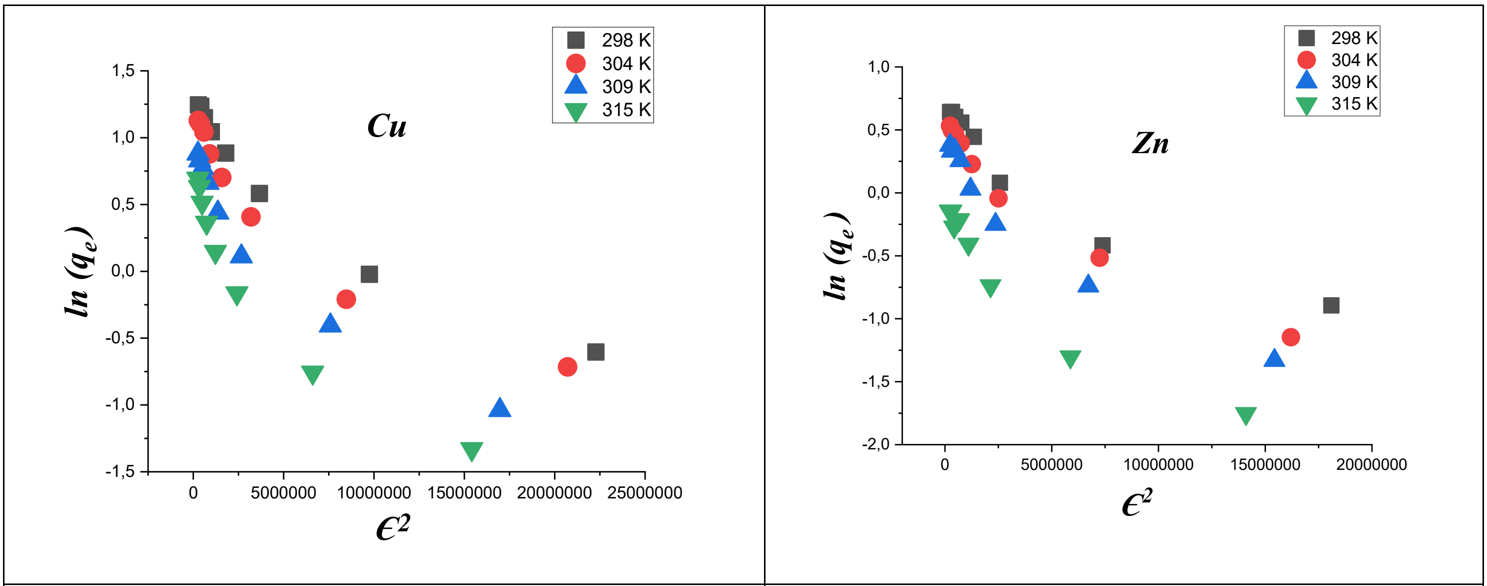

(e)	(f)

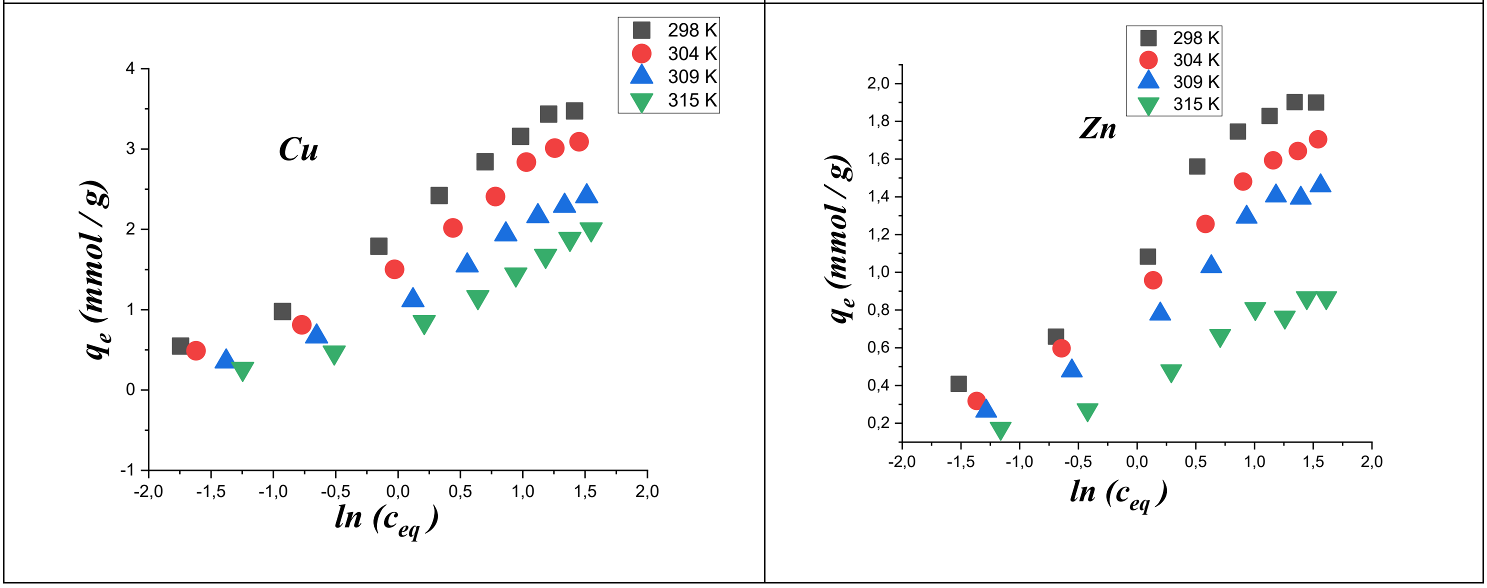

Figure S4. Freundlich plots for copper (a) and zinc (b). Dubinin-Radushkevich plots for copper (c) and zinc (d). Temkin plots for copper (e) and zinc (f).

Table S1. Kinetic parameters have been estimated by fitting the kinetic models with the experimental results.

Kinetic model	Model parameters	Copper	Zinc
Equilibrium sorption capacity	q_e	1.904	1.553
Pseudo -first- order	q_1	1.282	2.166
	K_1	0.024	0.028
	R^2	0.7825	0.9433
Pseudo-second-order	q_2	1.991	1.948
	K_2	0.0393	0.0099

	R^2	0.9977	0.9753
Weber Morris diffusion model	K_i	0.0627	0.1056
	Z	1.022	0.1169
	R^2	0.9578	0.9275
Elovich's equation	B	4.0064	2.385
	a	2.291	0.0854
	R^2	0.9786	0.9409
Dumwald-Wagner	K_{fd}	0.024	0.028
	R^2	0.7825	0.9433
Bangham kinetic model	K_γ	26.479	4.038
	γ	0.212	0.533
	R^2	0.9818	0.9496

Table S2. Freundlich, Dubinin-Radushkevich and Temkin parameters for copper

T (K)	*Freundlich parameters*			*Dubinin-Radushkevich parameters*				*Temkin parameters*			
	n	K_f	R^2	Q_S	K_{DR}	*E (kJ/mol)*	R^2	*B (kJ/mol)*	A_T *(L/g)*	$b*10^{-3}$	R^2
298	1.66	1.737	0.9758	3.555	2.00E-07	1.581	0.9812	2.318	2.476	1.068	0.9859
304	1.58	1.407	0.9871	3.166	2.00E-07	1.581	0.963	2.129	2.25	1.186	0.971
309	1.48	0.988	0.9854	2.511	3.00E-07	1.29	0.987	1.739	2.031	1.477	0.9768
315	1.35	0.6908	0.9961	1.983	4.00E-07	1.118	0.9571	1.487	1.809	1.76	0.9591

Table S3. Freundlich, Dubinin-Radushkevich and Temkin parameters for Zinc

T (K)	*Freundlich parameters*			*Dubinin-Radushkevich parameters*				*Temkin parameters*			
	n	K_f	R^2	Q_S	K_{DR}	*E (kJ/mol)*	R^2	*B (kJ/mol)*	A_T *(L/g)*	$b*10^{-3}$	R^2
298	1.86	0.989	0.9642	2.094	2.00E-07	1.58	0.9874	1.267	2.502	1.955	0.9687
304	1.73	0.807	0.9686	1.775	3.00E-07	1.29	0.9935	1.161	2.287	2.175	0.9912
309	1.63	0.647	0.9745	1.566	3.00E-07	1.29	0.9773	1.046	2.11	2.455	0.9772
315	1.63	0.373	0.9675	0.935	3.00E-07	1.29	0.959	0.634	2.024	4.125	0.9661
